# Decision Making under Ambiguity and Objective Risk in Higher Age – A Review on Cognitive and Emotional Contributions

**DOI:** 10.3389/fpsyg.2017.02128

**Published:** 2017-12-06

**Authors:** Magnus Liebherr, Johannes Schiebener, Heike Averbeck, Matthias Brand

**Affiliations:** ^1^Department of General Psychology: Cognition, University of Duisburg-Essen, Duisburg, Germany; ^2^Center for Behavioral Addiction Research, University of Duisburg-Essen, Duisburg, Germany; ^3^Erwin L. Hahn Institute for Magnetic Resonance Imaging, Essen, Germany

**Keywords:** aging, decision making, cognition, emotion, learning

## Abstract

The ability of decision making plays a highly relevant role in our survival, but is adversely affected during the process of aging. The present review aims to provide a better understanding of age-related differences in decision making and the role of cognitive and emotional factors in this context. We reviewed the literature about age-effects on decision-making performance, focusing on decision making under ambiguous and objective risk. In decisions under ambiguous risks, as measured by the Iowa Gambling Task, decisions are based on the experiences with consequences. In this case, many articles have attributed age-related impairments in decision making to changes in emotional and somatic reward- and punishment processing. In decisions under objective risks, as measured for example by the Game of Dice Task, decisions can be based on explicit information about risks and consequences. In this case, age-related changes have been attributed mainly to a cognitive decline, particularly impaired executive functions. However, recent findings challenge these conclusions. The present review summarizes neuropsychological and neurophysiological findings of age-related differences in decision making under ambiguous and objective risk. In this context, the relevance of learning, but also of cognitive and emotional contributors – responsible for age-related differences in decision making – are additionally pointed out.

## Introduction

Research on decision making over the life span shows fascinating, surprising and not seldom controversial results. Studies suggest that with increasing age people can display stability, improvements as well as downgrades when making decisions (Mata et al., 2011; Wiesiolek et al., 2014). On the one hand, older people have a large collection of experiences at their disposal, and seem to develop an emotional balance to make foresighted choices. On the other hand, some older people show a decline of cognitive functions. Other older persons demonstrate forgetfulness, inflexibility, slowness, and are overstrained when confronted with decisions. Many decision situations imply a risk that a bad choice is followed by suboptimal or very negative consequences. A psychological understanding of older adults’ decision-making competence and mechanisms contributing to risk-taking preferences are not only interesting from a basic-research perspective, but also from an applied perspective. Here, the increasing age of our world population – especially in the most industrialized countries – plays a highly relevant role. The literature provides some theoretical models, which describe neuropsychological mechanisms underlying decision making in general (Bechara et al., 1997; Brand et al., 2006; Schiebener and Brand, 2015a). However, there has been little attempts to bring together the diverse empirical findings and existing theoretical models to explain different changes in decision making of higher age.

The review aims at reviewing theoretical models on decision making as well as literature that investigated circumstances under which older aged individuals’ decision making undergoes changes and which mediators and moderators help to understand the underlying mechanisms. In contrast to previous theoretical works in the field of decision making, we focus especially on age related differences and implement both theoretical as well as empirical findings in a model at the end of the manuscript at hand.

## Two Types of Decision Situations and Two Exemplary Decision Tasks

In the present context, we address decision making under risk conditions, i.e., decision situations in which two or more options are available, the outcome of the decision is uncertain and there is a risk that suboptimal consequences follow, which cannot be completely anticipated (Kahneman and Tversky, 1979). For the review, we adopt the common distinction between decision making under ambiguous risk conditions and decision making under objective risk conditions (e.g., Krain et al., 2006; Schiebener and Brand, 2015a).

In decisions under ambiguous risk conditions there is no explicit information provided regarding the probabilities and extents of positive and negative consequences connected to the decision options (Brand et al., 2006). In other words, there are options available but individuals have no information they may use for reasoning which options are better and which are worse. Thus, they have to make choices and learn from feedback which options should better be preferred and which avoided. The task most frequently used to assess decisions under ambiguous risks is the Iowa Gambling Task (IGT; Bechara et al., 1994). The task has been used in uncounted numbers of studies addressing basic decision-making processes (e.g., Li et al., 2010), potential decision-making impairments in patients with neurological diseases and psychiatric disorders (e.g., Sevy et al., 2007), as well as studies on decision making and aging (e.g., Wiesiolek et al., 2014). In the IGT, participants are faced with four decks of cards lying face down. They have to choose between the four decks. After each choice, fictitious money is won but sometimes an additional loss follows. Participants have the aim to win as much money as possible and to lose as little of it as possible. During the course of the task participants can learn that the two left decks (A and B) lead to high gains but occasional very high losses. Overall, these two decks are disadvantageous. The two right decks (C and D) lead to low gains and occasional low losses. Overall, they are advantageous. The heights and occurrences of gains and losses vary in a way making it impossible to calculate probabilities. Most psychologically healthy individuals begin to prefer the advantageous options in the IGT on average around the 40th of the 100 IGT trials (Bechara et al., 1994; van den Bos et al., 2006). More details on the IGT can be found elsewhere (Buelow and Suhr, 2009).

In decisions under objective risk, there is explicit information about the rules for positive and negative consequences and the probabilities of their occurrence. The probabilities need not necessarily to be given but may also be calculable by considering the rules. In this type of decision situation individuals can make calculations to assess, which options are more favorable than others. In case the situation remains stable over several decision trials, individuals may even make long-term plans, develop strategies and apply them continuously. Two executive functions play a major role in generating a proper choice: (i) the ability to predict future outcomes of goal-directed actions; and (ii) the ability to cancel them when they are unlikely to accomplish valuable results (Mirabella, 2014). In fact, to make an optimal decision, the brain should confidently estimate the consequence of each choice. A very frequently used task to assess decision making under objective risk is the Game of Dice Task (GDT; Brand et al., 2005). Participants have to guess 18 times, which number will occur on top of a single virtual die. If they guess correctly, they win fictitious money, otherwise they lose the same amount of money. Participants can choose between 14 options involving four types of guesses: Betting on a single number or a combination of two, three, or four numbers. When they bet on more than one number they win, if one of the numbers in the combination is thrown. The possible gains and losses of the riskier options are higher (one number: €1,000, two numbers: €500, three numbers: €200, four numbers: €100). The winning probabilities are not presented but can be calculated (1/6, 2/6, 3/6, 4/6). Participants can calculate from the beginning that betting on less numbers is very risky and will probably lead to many high losses, while betting on more numbers is less risky and will probably lead to more frequent low gains and only low losses.

There are several other tasks assessing decisions under ambiguous risks [e.g., the Balloon Analog Risk Task (BART; Lejuez et al., 2002] or objective risks (e.g., the Cups Task, the Probability Associated Gambling Task, the Cambridge Gambling Task, and the Columbia Card Task; Figner and Voelki, 2004). In the present context, we mainly concentrate on the IGT and the GDT, respectively. Both tasks have been used in numerous previous studies to assess age-differences in decision making. Furthermore, the two tasks are not only used for addressing main effects of age, but also of cognitive and emotional factors. For example, Bruine de Bruin et al. (2012) reported a mediation of age and performance on decision tasks by fluid cognitive ability. Next to the IGT and GDT, other tasks will be mentioned if studies using them point to contrary results.

## Models of Decision Making and the Role of Age

Many models of decision making follow a dual-process approach (e.g., Epstein et al., 1996; Kahneman, 2003; Evans and Curtis-Holmes, 2005). In these models two systems are differentiated. One system is emotional, intuitive, impulsive as well as associative and works effortless, automatized and quickly. This system had the upper hand, when a decision was fast and thoughtless. It is – for example – called the impulsive system, intuitive-experiential system, or system one. The other system is rational, rule-guided, cognitively controlled and works effortful and slow. This system had the upper hand, when a decision was thought through, considering pros and cons, risks and chances and so on. In recent literature, dual-process models have been criticized for the strict separation of the systems, for potential theoretically wrong conclusions and are considered unsatisfactory by some authors (Evans and Stanovich, 2013, for review). From a neurobiological perspective there is strong evidence that the brain indeed has particular areas processing emotional impulses and particular areas processing cognitive reflections (Bechara, 2005). However, this evidence would also not support a strict separation of the systems but rather an interaction between them (Schiebener and Brand, 2015a).

There is a neurobiological oriented model on decision making suggested by Bechara (2005) implying an impulsive and a reflective system, which interact during the decision-making process. The impulsive system mainly involves the amygdala, ventral striatum and orbitofrontal cortex and is emotional and short-termly oriented. It elicits immediate emotional reactions (e.g., reward anticipation or fear) to the environment (e.g., presented decision options). The reflective system mainly involves the dorsolateral prefrontal cortex, anterior cingulate cortex and posterior parietal lobe and is long-term oriented. It processes knowledge and memories about possible consequences, situational rules and can control and strategize behavior.

The somatic marker hypothesis (e.g., Bechara and Damasio, 2005) harmonizes with this model. In summary, it says that making advantageous decisions can be learned emotionally from rewarding and punishing feedback from previous decisions. For example, when choosing a particular decision option is followed by a positive or negative consequence the impulsive system reacts with reward-processing, including the elicitation of bodily activation changes (e.g., increasing heart rate, visceral activations, slight muscle contractions, slight sweat segregation). The brain interprets these bodily reactions as being emotional. When being later on confronted with, the decision option may already be somatically marked. In this case, the reactions can be re-elicited, which can bias individuals’ cognitions and behaviors and guide individual toward the option again (in case it has been rewarding) or warn from choosing the option once more. These processes are anticipatory and can remain below an awareness threshold (Hicks et al., 2010). Somatic markers are regarded an important motivational aspect in decision making, providing individuals with affective information and the necessary emotional lift or warning in order to be able to make up their minds and be guided toward advantageous decision options. In contrast to the somatic marker hypothesis, Camille et al. (2004) and Coricelli et al. (2005, 2007) introduced a contrary perspective. The authors assume a top-down modulation of emotions as result of counterfactual thinking after a decision has been made (Camille et al., 2004; Coricelli et al., 2005, 2007). Furthermore, they reported reactivation of activity in the orbitofrontal cortex and amygdala occurring during the phase of choice, when the brain is anticipating possible future consequences of decisions. Based on these findings, Coricelli et al. (2007) suggested that the activation pattern reflects learning is based on cumulative emotional experience.

A more recent model (Schiebener and Brand, 2015a) follows the idea of two interacting systems and also implies age as a potentially modulating variable of decision-making processes. The model suggests that during decision making the impulsive and the reflective system are active but in most cases one of them is triggered as the leading processing mode. If this is the impulsive system, individuals go by immediate feelings (intuitions, impulses, urge for reward, fear of punishment), constituting a liking/disliking of options. If the reflective system guides the decision-making process, individuals use cognitive control (extract information, deliberate on options, plan, strategize and monitor behavior). In the case the decision is made under objective risk, this may also guide processing on ratios (e.g., calculating probabilities). In the impulsive system, feedback about consequences can trigger immediate reward and punishment reactions and can lead to the development of somatic markers. In the reflective system feedback can be used to check and monitor the success of a current decision-making strategy and revise the strategy (Brand et al., 2009). Whether a decision is made more impulsively or more reflectively is connected to the relative power of the two systems in a certain individual and situation and can lead to different decisions. For example, if the impulsive system has the upper hand decisions more probably become spontaneous and riskier. If the reflective system has the upper hand, decisions can become thought through, planned and guided by ratio considerations (Schiebener and Brand, 2015b). Which of the two processing systems becomes the leading one in a situation can be affected by several attributes of the individual and environmental aspects of the situation itself. For example, impulsive individuals and people in stress situations seem to be prone to be guided by the impulsive system, while persons with better executive functions or after induction of bad mood seem to be more frequently guided by the reflective system (Kahneman and Tversky, 1979; Epstein et al., 1996; Vohs, 2006; Schiebener and Brand, 2015a,b). Age is one of the factors named in the model that can affect impulsive and reflective processing in decision making, because aging has been shown to influence decision-making performance by affecting several executive functions and therefore our general ability of reasoning, processes controlled by reflective system. Given that we believe that alterations in cognitive abilities and emotional processing are the basis of age-related changes in decision making, we first consider the literature on the development of these aspects and then review the findings on age-related changes in decision-making performance.

## Alterations in Cognitive and Emotional Domains in Higher Age

The process of aging is accompanied by neuropsychological changes in cognitive and emotional domains. Corresponding with structural and functional brain changes – especially in the frontal lobe and the hippocampus (Fiell and Walhovd, 2010) – these changes typically affect executive functions such as inhibition, cognitive flexibility, planning, working memory, susceptibility to inference and strategy choice (Reuter-Lorenz and Sylvester, 2005; Uekermann et al., 2006; Allain et al., 2007; Ashendorf and McCaffrey, 2008; Elderkin-Thompson et al., 2008; Hodzik and Lemaire, 2011). In this context, Del Missier et al. (2012) discussed the ability to apply decision rules, and successful engagement in cognitive reflection as related to the monitoring and inhibition dimension of executive functions. In general, monitoring is described as key component for surviving in a constantly changing environment. This system is formed by a network of areas that determines the best strategy based on the available data, learned behaviors and the outcomes of previous actions. Depending on the task being performed, monitoring can engage different networks (Mirabella and Lebedev, 2017). Inhibition, as further executive function, must be seen as highly relevant in the context of impulse control in decision making. The relevance is witnessed by the wide range of neurological and psychiatric disorders characterized by poor control of urges such as Parkinson’s disease (e.g., Mirabella et al., 2012, 2017), eating disorders (Bartholdy et al., 2017), ADHD (Lipszyc and Schachar, 2010), gambling disorders (Marchetti et al., 2016; Nigro et al., 2018), OCD and depression (Christodoulou et al., 2006). Additionally, an age-related decline in the function of inhibition is reported in numerous previous studies (e.g., Sebastian et al., 2013; Bloemendaal et al., 2016; Coxon et al., 2016; Hsieh and Lin, 2017). Along with the function of monitoring and inhibition, Del Missier et al. (2012) discussed the executive function of shifting as important ability to provide consistent judgments in risk and is also adversely affected during the process of aging (e.g., Cepeda et al., 2001).

In the context of memory, many functions such as short-term memory, semantic memory and procedural memory remain relatively intact until old age (find summary in Glisky, 2007). A major difference between reduced vs. impaired functions is often seen in the amount to which they require active, quick and flexible cognitive processing that involves manipulation of information (see e.g., Salthouse, 1996; Salthouse et al., 2003). Thus, many older adults remain successful in the accomplishment of well-known everyday tasks and follow clearly instructed or familiar tasks (requiring semantic- and procedural memory) but are highly demanded when they need to combine new information, make plans or weigh up controversial pros- and cons (requiring quick information processing, cognitive flexibility, planning and/or working memory; see Glisky, 2007).

Emotional processing in older age has been reported to be biased in different ways compared to younger adults. Several authors have observed a positivity effect for processing of emotional information (Reed et al., 2014): for example, older adults are better at remembering positive information (Carstensen and Turk-Charles, 1994) and react less to negative stimuli (Knight et al., 2007), which indicates an insulation against negative information in higher age (Mather, 2012). This is accompanied by a reduction in amygdala activity during presentation of negative stimuli (Mather et al., 2004). Furthermore, older adults show an increase in prefrontal activity during presentation of emotional stimuli (Gunning-Dixon et al., 2003; Mather, 2012). Similar findings are reported in studies with more complex emotional stimuli such as pictures, words and faces. In comparison with patients with bipolar disorder, Altamura et al. (2016) identified that older adults recognized happy expressions faster and rated emotional faces more intensely. Further evidence comes from Mammarella et al. (2016b), who showed a higher sensitivity in older adults to positive stimuli by presenting a series of affective words or pictures. Within a second study of the research group (Mammarella et al., 2016a) the authors reported evidence for a potential involvement of different genetic polymorphisms in driving the positivity effect of older adults.

Event-related potential correlates of feedback processing have been observed to be less pronounced in older adults (Kardos et al., 2016). Comparable to the findings of the positivity effect, older adults adapt their behavior more to positive feedback and less to negative feedback (which tended to be the other way round in younger adults) (Di Rosa et al., 2015). In addition, activity in the ventral striatum positively correlated with age during rewarding feedback compared to neutral feedback (Vink et al., 2015). Rademacher et al. (2014) reported reduced activity in the nucleus accumbens of older adults when presenting monetary reward cues, while younger individuals showed increased activity (Rademacher et al., 2014). Samanez-Larkin et al. (2007) showed normal activity in striatal areas and the insular during gain anticipation in older age. In contrast, there was a relative reduction of activity during loss anticipation (see also Samanez-Larkin and Knutson, 2015). In reward anticipation, Vink et al. (2015) reported no general decline in activity during anticipation of consequences. Furthermore, Nielsen et al. (2008) reported increased negative arousal in younger adults when anticipating losses and positive arousal when anticipating gains, whereas older adults showed more positive arousal when anticipating gains but no increased negative arousal during the anticipation of losses.

In summary, studies focusing age-effects on emotion-processing, show consistent evidence that processing of negative information, negative feedback and loss/punishment are calmed in older adults. Positive information, positive feedback and gain/reward expectation were comparable to older adults or were intensified. Thus, there seems to be a negativity neglect combined with a tendency toward a positivity bias in several aspects potentially involved in decision making.

## Age-Related Changes in Decision-Making Performance

Mata et al. (2011), reviewed 29 studies, which considered older and younger individuals in tasks assessing decisions under ambiguous and objective risk. They observed that the pattern of age-related differences in decision making depend on the type of decision situation as well as the tasks used. Although, both the Balloon Analog Risk Task (BART) (participants had to choose between pumping up a balloon to earn more points if it doesn’t explode and collecting the earned money and getting a new balloon) and the IGT represent tasks for quantifying decision making under ambiguous risk, contrary findings between the two paradigm were reported (Mata et al., 2011, for review). In the IGT, older adults showed riskier and less advantageous behavior but when measured with the BART, older adults were more risk averse. Here, age-related effects were attributed to older-adults’ difficulties in learning from consequences. In decision making under objective risk, older adults behaved comparably to younger adults (e.g., in tasks offering a choice between a safe consequence and a gamble). However, when the decision task (such as the Cambridge Gambling Task) couples the low risk with low losses in the advantageous options and high risk with high possible losses in the disadvantageous options, older adults behaved less advantageously than younger adults (i.e., they had a higher preference for the high-gain–high-risk options). This conflict between high reward and risk may be particularly challenging for older adults (Mata et al., 2011) and is also inherent in the GDT.

In the following, we take a more detailed look at aging research. Here, we are focus on the IGT and GDT, which are considered among the most important decision-making tasks for decisions under ambiguous (IGT) and objective risk (GDT), respectively (Gleichgerrcht et al., 2010) (see **Table 1** for further details).

**Table 1:** Considered neuropsychological studies of age-related differences in decision making.

Studies	Participants		Task	Age-related differences	Explanations of the authors
	Younger	Older			
Bauer et al., 2013	*N*: 265, age: 23–88		IGT	Yes	Age-related increase in hypersensitivity to reward, whereby decisions of older adults are disproportionately influenced by prospects of receiving reward, irrespective of the presence or degree of punishment.
Beitz et al., 2014	*N*: 1,583, age: 5–89		IGT	Yes	Critical developments in decision processes during the adolescent years and decline in a cognitive process.
Brand and Schiebener, 2013	*N*: 538, age: 18–80		GDT	Yes	Relevance of executive functioning.
Carvalho et al., 2012	*N*: 40, age: 25.5 ± 4.7	*N:* 40, age: 67.4 ± 5.0	IGT	No	Significant differences in learning curve of the two age-groups.
Deakin et al., 2004	*N*: 177, age: 17–73		CGT	Yes	Age-related decreases in the risk tolerance factor, but unrelated to the delay aversion; neither factor was significantly related to verbal IQ.
Denburg et al., 2005	*N*: 40, age: 26–55	*N*: 40, age: 56–85	IGT	Yes	Disproportionate aging of the ventromedial prefrontal cortex.
Denburg et al., 2007	*N*: 40, age: 41.0	*N*: 40, age: 70.4	IGT	Yes	Poor decision makers display defective autonomic responses (or somatic markers).
Fein et al., 2007	*N*: 112, age: 37.8 ± 10.8	*N*: 52, age: 73.7 ± 7.4	IGT	Yes	Performance was associated with auditory working memory and psychomotor function in young adults, and immediate memory in older adults.
Kovalchik et al., 2005	*N*: 51, age: 18–36	*N*: 50, age: 70–95	IGT	No	Elderly individuals demonstrate highly accurate meta-knowledge evaluations. Older individuals have more accurate beliefs about their knowledge and its limitations.
Kovalchik and Allman, 2006	*N*: 65, age: 21.9 ± 3.9	*N*: 26, age: 80.5 ± 6.9	IGT	Yes	Equivalent monetary rewards might have less value to older adults than young adults, resulting in divergent preference behavior. Socioemotional selectivity theory which argues that an insensitivity to emotionally negative stimuli results from normative aging. Role of life experiences.
Lamar and Resnick, 2004	*N*: 23, age: 28.4 ± 5.9	*N*: 20, age: 69.1 ± 5.0	IGT	No	Sensitivity of the orbitofrontal cortex to age-related effects.
MacPherson et al., 2002	*N*: 30, age: 28.8 ± 6.0 *N*: 30, age: 50.3 ± 5.7	*N*: 30, age: 69.9 ± 5.5	IGT	No	Age-related differences depend on executive functions. Specific dorsolateral prefrontal theory of cognitive changes with age, rather than a global decline in frontal-lobe function.
Schiebener and Brand, 2017	*N*: 210, age: 18–86		IGT	Yes (last 60 trials)	Reductions in cognitive functions in older age.
Schneider and Parente, 2006	*N*: 42, age: 24 ± 4.4	*N*: 40, age: 68 ± 5.0	IGT	No	Lack of maintenance of the learning process.
Weller et al., 2011	*N*: 734, age: 5–85		Cups task	Yes	Evidence concerning the role of frontal lobe in decision making.
Wood et al., 2005	*N*: 88, age: 22.1 ± 4.5	*N*: 67, age: 77.3 ± 4.6	IGT	No	Different age-groups used different strategies. Strength of the younger group: learning and memory. Strength of the older group: accurate representation of wins and losses (valence).
Zamarian et al., 2008	*N*: 33, age: 36.1 ± 13.7	*N*: 52, age: 69.3 ± 7.0	IGT/PAG	Yes (IGT)/No (PAG)	Contribution of executive functions. Old people can make advantageous decisions when complete information about the decision situation is available.

Carvalho et al. (2012) provided new insight into the effects of learning in the context of age-related differences in decision-making under ambiguous risk. Although, they did not found overall differences between younger and older adults, they revealed significant differences between their learning curves. Considering single block-performance, older adults had a significantly better performance (only) in the first block compared to the younger ones. This is because the first block is the most implicit one and processing is guided by emotions, while the second is the most ambiguous block because there is no sufficient time to evaluate contingencies of gains and losses. No age-related differences neither in single block nor in overall IGT-performance were reported by Kovalchik et al. (2005) and Schneider and Parente (2006). In a further study, the authors pointed out the role of reversal learning in IGT-performance by using a modified version of the IGT, which involved a contingency reversal midway through the task (Kovalchik and Allman, 2006). Here, participants had to learn from recurrent changes of the decks from advantageous to disadvantageous. Thereby, reversal learning, the ability to adjust responses when the reinforcement value of stimuli change, is assumed to be distinct from the somatic marker process (Bechara et al., 2000) and affected by a decline of the ventromedial frontal cortex (Fellows and Farah, 2003). Fellows and Farah (2004) confirmed the assumption by indicating impairments in the IGT in both patients with ventromedial and patients with dorsolateral prefrontal lesions, but deficits of reversal learning were only shown in patients with ventromedial prefrontal abnormalities. The process of learning was further pointed out by Wood et al. (2005). Missing age-related differences in the IGT, the authors reported the usage of different strategies in younger and older adults. With an equal weight to gains and losses, they argue that older adults’ choices are highly dependent on learning parameter from recently experienced outcomes, rather than producing the maximum expected payoff. Bauer et al. (2013) used two versions of the IGT, in one version an immediate reward was always delivered regardless of deck choice while in the other version an immediate punishment was always delivered followed on occasion by a delayed reward. Age-related differences were only indicated in the first version. It is suggested that decision making in the elderly is disproportionally influenced by prospects of receiving reward, irrespective of the presence or degree of punishment. This is in turn in accordance with the socioemotional selectivity theory, which claims a fundamental role of time and therefore a change of social goals with a decrease of remaining time (Carstensen et al., 1999). Furthermore, Weller et al. (2011) reported a decrease in risk propensity with increasing age. This behavior is explained by the fact that older adults aim to achieve potential gains, rather than increased risk to avoid losses. Using the Cambridge Gambling Task, Deakin et al. (2004) reported, next to an age-associated reduction in risk-taking, longer deliberation times, poorer decision making, but no changes in delay aversion. Furthermore, the authors pointed out the relation between intelligence and the time need for the decision as well as the amount of modulation of risk-taking. While Denburg et al. (2005) indicated age-related differences in the total score of IGT-performance, they found no evidence in general cognitive functions such as attention, memory, visual perception or language, responsible for these differences. Only a weak relationship between used measures of cognition and IGT performance was reported by Beitz et al. (2014), although an interaction of modeling parameters suggested that cognitive changes are causal for age-related differences. Furthermore, Schiebener and Brand (2017) pointed out the role of cognitive abilities as mediator of age-related differences in both IGT and GDT performance. Thereby, age-related effects in the GDT were indicated only in the last 60 trials. Age-related differences in IGT were additionally associated with a decline in immediate but not delayed retrieve of memorized content (Fein et al., 2007). Again, Zamarian et al. (2008) reported an age-related decrease of IGT performance, whereas no differences were found in the Probability Associated Gambling task (PAG). In contrast to the IGT, decisions in the PAG task are based on estimable probabilities and alternatives, associated reward as well as punishments are explicitly given. Here, participants had to choose between a fixed amount of money or gamble in the lottery with the probability to win or lose a higher amount. Choosing the fixed sum means a gain or loss of €20, whereas choosing the lottery the participant will win €100 when a red cube is drawn, and lose €100 when a blue cube is drawn. Furthermore, participants were asked to perform tasks of executive functions such as phonological verbal fluency, categorical verbal fluency, verbal short-term memory, verbal working memory, divided attention, cognitive flexibility and mental complex calculation. Correlational analyses indicated a contribution of executive functions to both types of decisions. Similar findings are reported in the Game of Dice Task. For example, Brand and Schiebener (2013) indicated that people with good executive functions performed well in the GDT, whereas people with bad executive functions performed worse. Furthermore, the authors reported a mild correlation between age and decision making, moderated by subcomponents of executive functions (categorization, learning from feedback) and logical thinking (process of clearly moving from one related thought to another).

Another aspect discussed by Denburg et al. (2007) refers to autonomic responses. While a sizeable subset of older participants performed more disadvantageous in the IGT, the same poor decision-makers also displayed defective autonomic responses or somatic marker. Furthermore, the authors suggested a link between ventromedial prefrontal dysfunctions and decreased decision making. In contrast, MacPherson et al. (2002) postulated a greater sensitivity of orbitofrontal than ventromedial prefrontal cortex to the effects of aging. Using three cognitive tasks (WCST, Self-Ordered Pointed Task, Delayed-Response Task) assigned to dorsolateral prefrontal dysfunction and three (IGT, Faux Pas Task, Emotion Identification Task) to ventromedial prefrontal dysfunction, the authors reported age-related differences in dorsolateral but not in ventromedial prefrontal measures. Similar, Lamar and Resnick (2004) also assigned different measures either to the orbitofrontal (IGT, Delayed Match and non-match to sample task) or the dorsolateral prefrontal cortex (Self-ordered pointing task, Letter fluency, WAIT-R Digit Span Backward, Month Backward from the Boston Revision of the Wechsler Memory Scale). Here, the authors found no age-related differences in IGT performance, but proposed a sensitivity of measures of orbitofrontal cortex functioning to age effects.

In order to get a better understanding of age-related differences in neurophysiological findings, we end up the present section by reviewing imaging studies focusing older adults’ decision making (see **Table 2** for a summary). In this context, Samanez-Larkin et al. (2007) reported an age-related reduction of striatal activity in loss anticipation, but intact activity in gain anticipation. A relationship between increased variability in the nucleus accumbens and increased aging was reported in a further study by Samanez-Larkin et al. (2010), facing participants with financial decisions. Consistent with their behavioral results, which showed age-related impairments in learning from reward, Eppinger et al. (2013) demonstrated reduced ventromedial prefrontal activity during reward learning in the elderly. In the IGT, Rogalsky et al. (2012) reported an age-related increase in right ventromedial prefrontal cortex activity. Along with the ventromedial prefrontal cortex, Halfmann et al. (2016) reported a greater activity in the striatum during IGT performance in older adults. Thereby, the increased activity in prefrontal cortex was already reported in a previous study of the authors (Halfmann et al., 2014), where older adults showed more advantageous behavior in the IGT. Applying a two-choice prediction paradigm while participants were scanned with functional magnetic resonance imaging, Hosseini et al. (2010) reported a network of brain regions activated in healthy older adults similar to their younger counterparts. In contrast to others, the authors reported no increase in brain activity, but an age-related decrease in activity in the right inferior parietal lobule. Performing a risky-gains task older adults in the study by Lee et al. (2008) showed increased contralateral prefrontal activity, particularly in the orbitofrontal cortex as well as increased activity in the right insula in the older adults compared to the younger ones. The influence of the dopaminergic and serotoninergic brain system needs to be considered additionally. While Mohr et al. (2010) assumed a relationship based on the findings of decision making and neurotransmitter as well as aging and neurotransmitter, direct evidence comes from Chowdhury et al. (2013) (see also Shohamy and Wimmer, 2013). The authors used L-Dopa – the standard medication for Parkinson’s disease – to increase dopamine levels in the brain, in healthy older participants. Results demonstrated that increasing dopamine levels in the brain of the elderly increased task-based learning rate and task performance as well as activity in the striatum. Furthermore, Samanez-Larkin et al. (2011) reported that older adults with weaker correlations between activity in regions associated with the mesolimbic dopamine system and expected value, make less optimal decisions.

**Table 2:** Considered neurophysiological studies of age-related differences in decision making.

Studies	Participants		Task	Age-related differences	Underlying brain mechanisms
	Younger	Older			
Chowdhury et al., 2013	*N*: 22, age: 25.18 ± 3.85	*N*: 32, age: 70.0 ± 3.2	Two-armed bandit choice task	Yes	Age-related increase in dopamine level as well as activity in the striatum.
Eppinger et al., 2013	*N*: 13, age: 28.8 ± 3.3	*N*: 13, age: 70.0 ± 4.6	Two-choice decisions	Yes	Reduced ventromedial prefrontal activity during reward learning in the elderly.
Halfmann et al., 2014	*N*: 31, age: 59–88		IGT	Yes	Age-related increase in prefrontal cortex.
Halfmann et al., 2016	*N*: 80, age: 21	*N*: 29, age: 75.8 ± 6.8	IGT	Yes	Age-related increase in striatum activity.
Hosseini et al., 2010	*N*: 16, age: 20	*N*: 24, age: 69	Two-choice decisions	Yes	Age-related decrease in activity in the right inferior parietal lobule.
Lee et al., 2008	*N*: 12, age: 29.9 ± 6.2	*N*: 9, age: 65.2 ± 4.2	Risky-gains task	Yes	Age-related increase in contralateral prefrontal activity, particularly at the orbitofrontal cortex as well as the right insula.
Rogalsky et al., 2012	*N*: 15, age: 58–95		IGT	Yes	Age-related increase in right ventromedial prefrontal cortex activity.
Samanez-Larkin et al., 2007	*N*: 12, age: 19–27	*N*: 12, age: 65–81	MID	Yes	Age-related reduction of striatal and insular activity in loss anticipation.
Samanez-Larkin et al., 2010	*N*: 54, age: 21–85		Dynamic financial investment task	Yes	Age-related increase in variability in nucleus accumbens activity.
Samanez-Larkin et al., 2011	*N*: 12, age: 19–26	*N*: 13, age: 63–85	Intertemporal decision making task	Yes	Relevance of mesolimbic dopamine system as well as striatal regions during the process of aging.

In summary, evidence from both behavioral and neurophysiological studies highlighted the effects of the process of aging on humans’ decision-making.

## Bringing Together the Theoretical Models and Empirical Findings

The present review was conducted in order to provide a better understanding of decision making under ambiguity and objective risk in the elderly. On the one hand, we confirm the findings of the key role of learning (Mata et al., 2011; Samanez-Larkin and Knutson, 2012). On the other hand, we suggest a lot more variables – adversely affected during the process of aging – responsible for characterizing older adults’ decision making.

**FIGURE 1:**
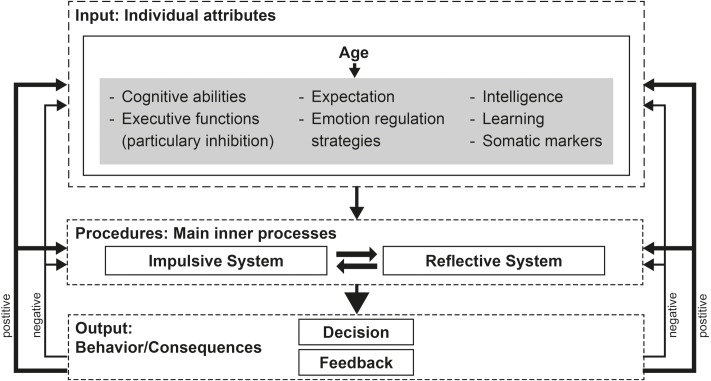
Modified version of the decision-making model of Schiebener and Brand (2015a). The illustrations give an overview of aspects, relevant in older adults’ decision making. Individual attributes (with the gray background) should not be seen as disjoint constructs, but rather as overlapping and interacting functions.

In order to get a better understanding of the variables – responsible in this context – we modified the model proposed by Schiebener and Brand (2015a) (see **Figure 1**).

In the original model Schiebener and Brand (2015a) pointed out three aspects named ‘individual attributes,’ ‘information about the decision situation,’ and ‘situational induced states and external influences’ as input factors, affecting the process of decision-making. Due to the fact that the present modified model focus exclusively on age-related differences, we disregarded the external factors ‘information about the decision situation’ and ‘situational induced states and external influences,’ which are not influenced by the process of aging. Furthermore, we disregarded the individual attributes ‘need for arousal,’ ‘state impulsivity,’ and ‘self-control,’ which have not been reported to be influenced in elderlies’ decision making. In this context, it should be noted that these factors are related to the function of inhibition, summarized under the term of executive functions. We added the variables of learning, intelligence and expectation, which are described to be influenced during the process of aging. In sum, there are numerous individual attributes affected during the process of aging. Some of them act as mediator/moderator and affect the process of decision making. These attributes comprising cognitive abilities (such as visual perception, and language), somatic marker, expectation (e.g., the ability to predict future outcomes), emotion regulation, intelligence, learning and executive functions.

The considered studies reported highly inconsistent effect-sizes of considered variables. Within the model we integrated all aspects without any weight or priority, as well as hierarchical structure. We argue that the inconsistency is based on numerous factors. First, the mean age of sample size differs strongly between the single studies from 69.1 years (Lamar and Resnick, 2004) to 82 years (Kovalchik et al., 2005) (see also **Tables 1**, **2**). Second, the use of the paradigm might also influence the effect size. While most IGT studies focused the total score, some reported age-related differences in a single-block consideration. Carvalho et al. (2012) for example found no overall differences between younger and older adults in IGT performance but found a significantly better performance in the first block of the elderly. Furthermore, Beitz et al. (2014) indicated a correlation of Wisconsin Card Sorting Test, *n*-back task and matrices subtests performance with IGT decks C + D but not with B + D. Third, effects of task characteristics additionally influence age-related differences in decision making. This became obvious in the study conducted by Bauer et al. (2013) who reported age-related differences in the condition of the IGT, which requires choosing lower immediate reward but not in the condition, which requires choosing higher immediate punishment.

Within the second step of the model named ‘procedures: main inner processes,’ we suppose an age-related influence of both, the impulsive and the reflective system. While the original model describes the impulsive system as consisted of emotional reactions, conditioning as well as somatic activity, the reflective system is described to be associated with executive functions and working memory. The considered studies indicated age-related impairments in components of both systems. Furthermore, neurophysiological findings demonstrated reduced activity in the striatum as well as the orbitofrontal cortex – mainly involved in impulsive decisions – of older adults as well as age-related differences in dorsolateral prefrontal cortex and the parietal lobe, which are – inter alia – associated with reflective decisions. Considering unfamiliar situations in which people had to analyze, balance, plan etc., executive functions especially working memory capacity might be highly relevant. Therefore, it could be assumed that handling these situations are affected during the process of aging. In contrast, we suppose that experiences from the past such as strategies of risk-avoidance are used as compensational strategies. This is in accordance with the findings that long-term memory, procedural memory, etc. are relatively unaffected in the elderly. Furthermore, it could be assumed that successful processing might also depend on the amount of crystalline or fluid intelligence used in the respective situation (see also Li et al., 2013).

In the last step of the decision-model by Schiebener and Brand (2015a) named ‘Output: Behavior/Consequences,’ we further differentiate between positive and negative feedback. While the original model didn’t consider a subdivision of feedback, this aspect might be highly relevant in older adults’ decision making. As already stated in the previous subchapter, older adults tend to hide negative feedback/information, whereas positive feedback/information is intensified. This is underpinned by the fact that feedback processing about reward and punishment as well as anticipating reward and punishment in decision making are major emotional components (e.g., Bechara et al., 1994, 1997; Damasio, 1994; Figner et al., 2009; Figner and Murphy, 2011; Panno et al., 2013; Schiebener and Brand, 2015a) and adversely affected during the process of aging.

## Conclusion

The present review demonstrates the importance of considering decision making in older adults. Until now there is a limited number of studies focusing the effects of different cognitive and emotional mediator or moderator. Furthermore, existing studies in this context are highly inconsistent, which lead to difficulties in comparing the results. There is also a lack of longitudinal studies. Nevertheless, the review at hand provided an overview of possible variables affecting older adults’ decision making as well as a possible assignment in this context. We pointed out the relevance of learning, but further addressed cognitive and emotional contributors, responsible for age-related differences in decision making. Based on these findings, future studies should systematically focus on possible mediators and moderators affecting decision making in the elderly.

## Author Contributions

All authors listed have made a substantial, direct and intellectual contribution to the work, and approved it for publication.

## Conflict of Interest Statement

The authors declare that the research was conducted in the absence of any commercial or financial relationships that could be construed as a potential conflict of interest.
